# Are fish immune systems really affected by parasites? an immunoecological study of common carp (*Cyprinus carpio*)

**DOI:** 10.1186/1756-3305-4-120

**Published:** 2011-06-27

**Authors:** Karolína Rohlenová, Serge Morand, Pavel Hyršl, Soňa Tolarová, Martin Flajšhans, Andrea Šimková

**Affiliations:** 1 Department of Botany and Zoology, Faculty of Science, Masaryk University, Kotlářská 2, 611 37 Brno, Czech Republic; 2 University of Montpellier 2, CNRS-IRD, Institute of Evolutionary Sciences, CC065, 34095, Montpellier, France; 3 Institute of Experimental Biology, Faculty of Science, Masaryk University, Kotlářská 2, 611 37, Brno, Czech Republic; 4 University of South Bohemia České Budějovice, Research Institute of Fish Culture and Hydrobiology in Vodňany, Zátiší 728/II, 389 25 Czech Republic

## Abstract

**Background:**

The basic function of the immune system is to protect an organism against infection in order to minimize the fitness costs of being infected. According to life-history theory, energy resources are in a trade-off between the costly demands of immunity and other physiological demands. Concerning fish, both physiology and immunity are influenced by seasonal changes (i.e. temporal variation) associated to the changes of abiotic factors (such as primarily water temperature) and interactions with pathogens and parasites. In this study, we investigated the potential associations between the physiology and immunocompetence of common carp (*Cyprinus carpio*) collected during five different periods of a given year. Our sampling included the periods with temporal variability and thus, it presented a different level in exposure to parasites. We analyzed which of two factors, seasonality or parasitism, had the strongest impact on changes in fish physiology and immunity.

**Results:**

We found that seasonal changes play a key role in affecting the analyzed measurements of physiology, immunity and parasitism. The correlation analysis revealed the relationships between the measures of overall host physiology, immunity and parasite load when temporal variability effect was removed. When analyzing separately parasite groups with different life-strategies, we found that fish with a worse condition status were infected more by monogeneans, representing the most abundant parasite group. The high infection by cestodes seems to activate the phagocytes. A weak relationship was found between spleen size and abundance of trematodes when taking into account seasonal changes.

**Conclusions:**

Even if no direct trade-off between the measures of host immunity and physiology was confirmed when taking into account the seasonality, it seems that seasonal variability affects host immunity and physiology through energy allocation in a trade-off between life important functions, especially reproduction and fish condition. Host immunity measures were not found to be in a trade-off with the investigated physiological traits or functions, but we confirmed the immunosuppressive role of 11-ketotestosterone on fish immunity measured by complement activity. We suggest that the different parasite life-strategies influence different aspects of host physiology and activate the different immunity pathways.

## Background

Physiology and immunity in fish, a group of poikilothermic vertebrates, are strongly influenced by both abiotic and biotic factors. Water temperature is generally considered as the strongest abiotic factor which affects fish physiology including immune functions. However, the infection dynamics of fish parasites and pathogens is also strongly influenced by water temperature changes [1,2].

To determine whether the observed status of fish physiology results from abiotic changes or reflects the level of parasite infestation is very difficult in natural conditions because of the confounding effects of several abiotic and biotic factors including parasitism, often varying in space and time. Recently, many studies have focused on the abiotic effects, especially of water temperature, on physiological and immunological mechanisms in poikilothermic organisms, like fish. The majority of immunological studies have suggested an immune-suppression effect associated with a decrease in water temperature [3-7]. Moreover, the immunosuppressive effects of polychlorinated biphenyls are known in fish species [e.g. [8,9]]. Teleost fish possess similar immune system mechanisms to mammals - both non-specific (innate or natural) and specific (acquired or adaptive) [10]. However, substantial differences exist between the immune systems of poikilo- and homoiothermic organisms. According to Ainsworth et al. [11], the specific branch of immunity is more sensitive than the non-specific defence at lower water temperature, and was assumed to be more important for poikilothermic than for homoiothermic vertebrates [12]. Moreover, Le Morvan et al. [5] suggested that, at low water temperature, the non-specific defence of fish immune system tends to offset specific immune suppression until the specific immune system adapts.

Several studies have reported that the decrease in water temperature may cause the suppression of acquired immunity, with the components of innate immunity being relatively independent of water temperature [13]. Other seasonally-dependent events like spawning in fish could more strongly influence immunity than water temperature [14]. However, many studies have also shown how water temperature drives the seasonal changes in parasite infection, mainly because parasite reproduction and survival of free-living infective stages of parasites are dependent on a specific range of temperature [15].

Close interactions occur likewise between fish host and parasites. The interactions between fish physiology (associated with host size, age, sex etc.) and the level of parasite infection have been relatively well documented [16,17]. However, there have been few studies investigating the effects of seasonal changes on selected measures of host physiology in relation to parasite load [e.g. [18]], and fish immune response has mostly been studied solely in relation to parasite infection [19,20].

The contribution of immunoecological studies has gained a place of central importance [21,22]. Life-history theory is at the core of immunoecological studies. The principle assumption is that each organism has a limited amount of energy which is allocated to different fundamental functions (i.e. maintenance including immune defence, reproduction and growth) in accordance with current needs [23]. The activation of the immune system is energetically costly [24,25]. Therefore, trade-offs are expected to occur as hosts infected by parasites should invest energy into immune responses, at the expense of other physiological tasks [26]. However, optimum host defence is governed by a parasite-mediated allocation trade-off between growth and immune function (see Tschirren and Richner [27]).

Furthermore Folstad and Karter [28] introduced the immunocompetence handicap hypothesis, which predicts that the steroid hormones responsible for the production of sexual signals in males may cause immunosuppression especially during the reproductive period. Many recent studies have been conducted to test whether the expression of sexual ornamentation is associated to immunosuppression in fish [e.g. [29,30]].

The aims of the present study were to analyze the effects of seasonal changes (i.e. temporal variability) on (a) selected physiological and immune parameters and on (b) the infection by metazoan parasites. We examined the potential associations between immunity, physiology and parasite infection following the assumption of trade-offs in energy allocation using the total investment in immunity, physiology and total measure of parasitism as well as analyzing each measure of immunity and physiology separately. We tried to estimate whether the seasonal changes of abiotic environment are the main driver of immune activation in order to face risks of being parasitized or whether the observed associations between immunity and physiology are the results of trade-off without being affected by seasonal changes. Finally, we tested the hypothesis of immunosuppression by 11-ketotestosteron in fish males.

## Methods

### Fish sampling

A total number of 160 three-to-four-year-old individuals of common carp (*Cyprinus carpio *Linnaeus 1758, Cyprinidae), including 87 males and 73 females were collected from a pond-farmed population (Vodňany, South Bohemia, Czech Republic) in five selected months in 2007 and 2008. Each sample represented a different season and diverse water temperature, i.e. June 2007 - early summer (16°C), August 2007 - late summer (18.4°C), November 2007 - autumn (4.9°C), February 2008 - winter (2.8°C) and April 2008 - spring (7.5°C). The temperature on the day of collection was measured. The fish were sampled using seine netting and then separated according to their sex. Samples of blood were taken immediately from each specimen from the caudal vein using heparinized syringes following Pravda and Svobodová [31]. Blood was preserved in microtubes with heparin (50 U/ml, Zentiva). Blood samples used for measuring oxidative burst activity and haematology (differential leukocyte counts and total leukocyte counts) were processed immediately after blood collection. Blood samples used for other immunological analyses (complement activity, IgM) and 11-ketotestosterone concentration were deep-frozen at -80°C.

Each fish individual was intramuscularly tagged for later identification using a P.I.T. tag (134.2 kHz, AEG ID-162, AEG Co., Ulm, Germany) in the left side of the dorsal part close to the first hard dorsal fin ray. Fish were transported to the laboratory in tanks containing original water from the pond. Fish were killed by severing the spine. Each individual was measured (total length in centimetres) and weighed (in grams). A complete parasitological dissection for all metazoan parasites was immediately performed for 145 individuals (including 80 males and 65 females) according to Ergens and Lom [32].

### Respiratory burst activity

Phagocytes (granulocytes and monocytes/macrophages) are considered to be the first line of immune defence against pathogens overcoming the natural barriers. These cells have the ability to engulf and kill pathogens during the so-called "oxidative burst" leading to production of reactive oxygen species [33]. Seasonal changes in phagocytic activity have been studied in different fish species [34,11].

Respiratory burst activity was measured as luminol- enhanced chemiluminescence using a luminometer (LM01-T, Immunotech, Czech Republic) and opsonised Zymosan A as activator [35,36]. The maximal intensity of respiratory burst (peak in relative light units - RLU) was evaluated in this study.

### The measurement of complement activity in plasma

Among other non-specific humoral factors (i.e. non-cellular defence mechanisms) complement system plays an important role in natural defence against pathogens. Complement contains a series of serum proteins that are activated using either a classical (antibody dependent) or alternative pathway. Complement participates in lytic, pro-inflammatory, chemotactic and opsonic activities, thus it forms the connection between non-specific humoral and cellular mechanisms (i.e. phagocyte responses) [37]. One of the most important and well known complement functions is the capacity to create pores in the membrane of the pathogens' surface and thereby kill them. Hernández et al. [7] reported a close relationship between water temperature and the level of complement activity. The role of complement in monogenean infection was demonstrated in salmonid fish [38,39].

Complement activity was measured according to Virta et al. [40] and Nikoskelainen et al. [41] with modifications. The total complement activity (including all activation pathways) of plasma was determined using a bioluminescent strain of *Escherichia coli *(K12luxAmp, kindly provided by University of Turku, Finland). The light emission measured by LM01-T luminometer was positively correlated with the viability of *E. coli*. The relative measure of complement activity was estimated by computing the difference between the maximal time of measurement (equal to 4 h) and the time necessary for killing 50% *E. coli *by complement (in h). For the details see Buchtíková et al. [42].

### The determination of total IgM level

The major component of fish specific humoral defence is immunoglobulin M (IgM), although IgD [43] and even IgZ and IgT [44,45] have also been recently described. Clear seasonal changes of plasma IgM levels were found to be related to water temperature and/or gonad maturation [46]. The production of specific immunoglobulin against gill monogeneans [16] or other helminths [47] was observed. Specific antibodies play an essential role in cytolytic or cytotoxic mechanisms, such as in the activation of the complement system (classical pathway) or helping leukocytes to adhere to the parasite surface, presumably through Fc-like receptors [48].

The total IgM level was determined using precipitation with zinc sulphate (0.7 mM ZnSO_4_.7H_2_O, pH = 5.8) [49]. The concentration of IgM in the sample (in g/l) was calculated as the difference between total proteins (commercial kit, Bio-Rad, USA) and proteins in the supernatant after precipitation and centrifugation.

### 11-ketotestosterone level

The immune system is affected by the level of steroid hormones. The 11-ketotesterone (11-KT) is a major androgen in the majority of teleost fish, responsible for sexual behaviour and spermatogenesis, found in higher levels in the blood plasma or serum in males than in females [50]. As already mentioned, these hormones have an immunosuppressive effect. The level of 11-ketotestosterone in male plasma was analyzed following the protocol provided in the commercial competitive enzyme immunoassay kits (Cayman Chemical, Estonia). For the details see Buchtíková et al. [42].

### Haematological analyses

The determinants of white-blood cell count (including leukocyte and lymphocyte counts, and differential leukocyte counts) are considered to be an important parameter of fish health status. Like other haematological parameters, white-blood cell counts depend on various abiotic and biotic factors such as water temperature, environmental stress, fish sex and age [51,52]. According to Ruane et al. [53], fish infected by parasites significantly changed their haematological parameters. Leukocyte counts can be applied as a measure of general immune response. The increased leukocyte counts and shift values towards the myeloid line (especially a high number of myelocytes and metamyelocytes) reflect the current infection or inflammation [54].

Leukocyte counts (in g/l) were determined according to the methodology of Svobodová et al. [55] and Lusková [56] using Natt-Herrick solution. A differential leukocyte profile was assessed following Lamková et al. [18]. We estimated the percentage distribution of all types of white blood cells. However, we used only the leukocyte count and the relative count of lymphocytes and phagocytes (in g/l) for statistical analyses (see Rohlenová et al. [30]). Finally, haemoglobin content (Hb) was analyzed photometrically (540 nm; Helios Unicam, USA) in Kampen-Zijlster transformation medium.

### Spleen size

The spleen, the thymus, but also the head and trunk of the kidney belong to the principal lymphomyeloid tissues of teleosts. The spleen as a secondary lymphatic and scavenging organ plays an important role in haematopoiesis, antigen degradation and antibody production processing [57]. This organ is also known to act as an erythrocyte reservoir [58]. The spleen size of fish is widely used as a simple measurable immune parameter with a potential role in immune response against parasite infection [59-61]. A link between spleen size and fish condition was also previously documented [62]. In this study, we measured spleen weight (in grams at accuracy 0.001 g). The spleen-somatic index (SSI) was calculated as spleen weight (g)/body weight (g) × 100.

### Other physiological parameters

We measured liver and gonad weight (in grams). We calculated the relative body weight (i.e. condition factor, K) using the equation: K = constant × somatic weight (g)/(standard length [cm])^3 ^according to Bolger and Connolly [63]. The relative size of gonad (i.e. gonado-somatic index, GSI) was calculated as follows: GSI = gonad weight (g)/body weight (g) × 100, and the relative size of liver (i.e. hepato-somatic index, HSI) was calculated as follows: HSI = liver weight (g)/body weight (g) × 100.

### Parasite collection and determination

Fish hosts were investigated for all metazoan parasites (Table 1). Collected parasites were determined using recent keys [64-68]. Due to the very high parasite abundance on fish gills and because *Dactylogyrus *(see Table 1), in particular, die quickly after the killing of the fish, we collected these parasites only from the right side of the gills following Kadlec et al. [69]. For the details on parasite fixation and identification see Lamková et al. [18]. Epidemiological characteristics such as prevalence (percentage of infected host individuals in each sample), intensity of infection (number of parasites per infected host), and abundance (mean number of parasites per host individual in each seasonal sample) were calculated for each parasite species according to Bush et al. [70].

**Table 1:** Parasite abundance, intensity of infection and prevalence

		Abundance ± SD	Intensity of infection (min-max)	Prevalence (%)	Abundance ± SD	Intensity of infection (min-max)	Prevalence (%)	Abundance ± SD	Intensity of infection (min-max)	Prevalence (%)
Parasites	Parasite species	Early summer			Late summer			Autumn		

Monog	*D. molnari *Ergens & Dulma, 1969	79.04 ± 54.61	6-204	100	1065.63 ± 592.69	267-2450	100	1816.62 ± 1623.86	159-6879	100
	*D. extensus *Mueller & Van Cleave, 1932	16.33 ± 9.55	1-40	100	179.39 ± 132.67	4-611	100	99.95 ± 222.96	0-1212	97
	*D. falciformis *Achmerow, 1952	7.16 ± 8.77	0-32	84	63.9 ± 46.05	3-167	100	207.54 ± 156.32	30-528	100
	*D. achmerowi *Gussev, 1955	3.55 ± 5.08	0-20	72	36.21 ± 21.65	0-81	97	46.16 ± 35.16	0-133	97
	*D. anchoratus *(Dujardin, 1845)	0.08 ± 0.28	0-1	8	2.3 ± 3.82	0-12	30	1 ± 3.93	0-19	7
	*Gyrodactylus *spp.	0.88 ± 1.9	0-8	28	-	-	-	0.8 ± 1.32	0-4	33
	*Eudiplozoon nipponicum *(Goto, 1891)	8.44 ± 4.08	2-20	100	22.77 ± 17.45	0-53	97	1.1 ± 1.63	0-7	47
Crusta	*Argulus foliaceus *(Linnaeus, 1758)	13.4 ± 11.22	3-47	100	9.27 ± 7.17	0-27	87	8.57 ± 5.79	0-25	97
	*Ergasilus sieboldi *Nordmann, 1832	0.04 ± 0.2	0-1	4	-	-	-	0.07 ± 0.25	0-1	7
Cesto	*Antractolytocestus huronensis *Anthony 1958	6.24 ± 15.30	0-56	28	472.83 ± 964.82	0-5014	83	19.73 ± 36.26	0-119	43
	*Khawia sinensis *Hsü, 1935	0.16 ± 0.55	0-2	8	-	-	-	0.5 ± 1.43	0-6	17
	*Valipora campylancristrota *(Wedl, 1855)	-	-	-	0.17 ± 0.65	0-3	7	-	-	-
Dige	*Diplostomum *larv sp.	5.28 ± 6.94	0-31	68	1.97 ± 3.50	0-12	33	6.13 ± 7.74	0-31	73
Moll	Glochidium spp.	-	-	-	0.07 ± 0.37	0-2	3	-	-	-
Hirud	*Piscicola geometra *(Linnaeus, 1761)	0.04 ± 0.2	0-1	4	-	-	-	0.13 ± 0.43	0-2	10

		Winter			Spring					

Monog	*D. molnari *Ergens & Dulma, 1969	81.72 ± 144.99	0-825	97	229.56 ± 662.57	23-3653	100			
	*D. extensus *Mueller & Van Cleave, 1932	1.25 ± 2.55	0-9	33	2.59 ± 7.08	0-38	59			
	*D. falciformis *Achmerow, 1952	3.99 ± 12.15	0-67	67	30.58 ± 148.64	0-803	86			
	*D. achmerowi *Gussev, 1955	12.69 ± 30.36	0-161	90	12.92 ± 31.04	0-172	97			
	*D. anchoratus *(Dujardin, 1845)	-	-	-	-	-	-			
	*Gyrodactylus *spp.	303.83 ± 1094.16	0-5664	70	2.14 ± 2.29	0-8	66			
	*Eudiplozoon nipponicum *(Goto, 1891)	0.43 ± 0.86	0-3	27	0.3 ± 0.53	0-2	33			
Crusta	*Argulus foliaceus *(Linnaeus, 1758)	-	-	-	0.03 ± 0.19	0-1	3			
	*Ergasilus sieboldi *Nordmann, 1832	0.03 ± 0.18	0-1	3	-	-	-			
Cesto	*Antractolytocestus huronensis *Anthony 1958	-	-	-	-	-	-			
	*Khawia sinensis *Hsü, 1935	1.37 ± 3.52	0-15	20	0.62 ± 1.40	0-6	24			
	*Valipora campylancristrota *(Wedl, 1855)	0.03 ± 0.18	0-1	3	-	-	-			
Dige	*Diplostomum *larv sp.	3.7 ± 4.02	0-16	80	4.05 ± 3.43	0-14	86			
Moll	Glochidium spp.	-	-	-	-	-	-			
Hirud	*Piscicola geometra *(Linnaeus, 1761)	0.03 ± 0.18	0-1	3	-	-	-			

Parasite abundance (mean with standard deviation), intensity of infection (minimum and maximum values) and prevalence for each metazoan parasite species found on common carp collected within each sampled period. Monogenea (Monog), Crustacea (Crusta), Cestoda (Cesto), Digenea (Dige), Mollusca (Moll), and Hirudinea (Hirud).

### Statistical analyses

Most of the measured parameters did not fit a normal distribution and required transformation logarithm, hyperbolic arcsine, hyperbolic arcsine square root or square root transformations. First, preliminary analyses testing the effects of season and sex on parasitism, physiology and immunity were performed using one-way ANOVAs.

Following Poisot et al. [71], we used an indicator of parasite community structure based on principal component analysis (PCA) that takes into account all parasite species and the number of individuals for each parasite species within host individuals. In the same manner, we used an indicator of immunity based on PCA that takes into account all immune variables (spleen-somatic index, leukocyte, lymphocyte and phagocyte counts, IgM level, respiratory burst and complement activity). Finally, an indicator of physiology was also based on PCA that takes into account parameters linked to physiology (condition factor, gonado-somatic index, hepato-somatic index and haemoglobin).

We extracted values of the first three principal components (PCs) of each of these PCAs (i.e. principal components having eigenvalues over one were considered following Vainikka et al. [72]). These PCs represent a measure of parasitism (PCs of parasites), a measure of immunity (PCs of immune variables) or a measure of physiology (PCs of physiological variables) respectively. Then, we performed Pearson correlation between sampling period and all three PCs for each parasitism, immunity and physiology. Further, partial correlations controlling for sampling period were performed to analyze the potential relationships between parasitism, immunity and physiology. Next, GLM analyses were performed to investigate the potential associations between parasite abundance and all immune and physiological variables taking into account the sampling period. Last, GLM analyses were conducted to analyze the associations between immune and physiological variables taking into account the effect of sampling period or alternatively the effects of both sampling period and sex following the preliminary analyses. As the spleen, gonad and liver weights were correlated with total body weight; we used SSI, GSI or HSI. All statistical analyses were executed using Statistica 9.0 for Windows.

## Results

### Seasonal changes of parasite infection and gender differences

The basic characteristics of parasite infection were estimated for each parasite species within each sampling period (Table 1). The metazoan parasites belonging to six parasitic groups were found on common carp including ectoparasitic Monogenea, Crustacea, Mollusca and Hirudinea, and endoparasitic Cestoda and Digenea. No Nematoda or Acanthocephala were observed. Monogenea was the species' richest and most numerous group (almost 90% of total parasite abundance) and included *Eudiplozoon nipponicum*, five *Dactylogyrus *species (four of them present in all sampling periods) and viviparous *Gyrodactylus *species. In addition, three species of Cestoda, two species of Crustacea, one species of Hirudinea and one species of Digenea were found. The larval stages of Mollusca were undetermined. Following the variability in abundance among the various metazoan parasites (see Table 1), only the parasite groups with high abundance were included in statistical analyses i.e. Monogenea as the most abundant group, Crustacea and Cestoda characterized by the presence of one dominant species and one or two rare species, and Digenea represented only by larval stages of *Diplostomum *species. Due to low abundance Hirudinea and Mollusca were not included into statistical analyses.

**Figure 1:**
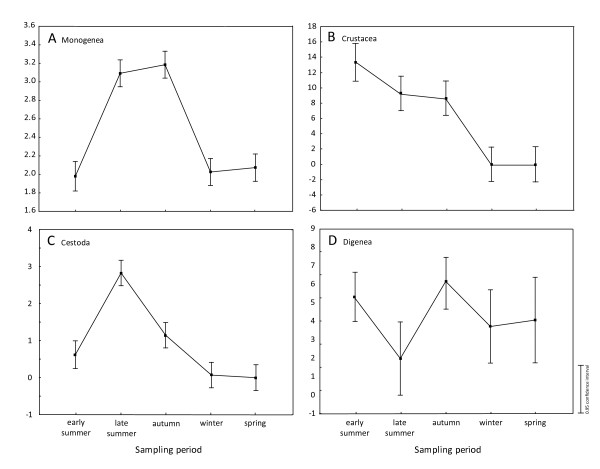
**The changes in parasite abundance in relation to sampling period**. Detailed legend: The changes in abundance of (A) Monogenea, (B) Crustacea, (C) Cestoda and (D) Digenea in relation to sampling period. Log transformation for abundance of Monogenea and hyperbolic arcsine square root transformation for Cestoda were applied.

Using one-way ANOVA, significant effects of sampling period were observed on the abundance of Monogenea (F_4,139 _= 66.951, p < 0.0001), Cestoda (F_4, 139 _= 44.108, p < 0.0001) and Crustacea (F_4, 139 _= 25.707, p < 0.0001). A marginal but significant effect of sampling period on the abundance of Digenea was found (F_4, 132 _= 2.488, p = 0.046). Clear patterns emerge for Monogenea with the highest values of abundance observed in late summer and autumn (Figure 1A) due to peak infection of *Dactylogyrus. D. molnari*, in particular, reached extremely high abundance. Viviparous *Gyrodactylus *species were present only in winter (in very high abundance) and spring (Table 1). Abundances in Crustacea decreased from early summer to spring (Figure 1B) resulting from the seasonal variation of *Argulus foliaceus *(see Table 1). Cestoda reached highest abundance in late summer (Figure 1C) resulting from the seasonal variation of *Antractolytocestus huronensis *(see Table 1). Only a slight seasonal variation in abundance of larval Digenea was found (Figure 1D). ANOVA showed a weak significant effect of host sex only on the abundance of Cestoda (F_1, 142 _= 4.1041, p = 0.045) with higher abundance values recorded in females.

### Seasonal effect on host physiology and immunity and gender differences

All measured variables were influenced by the sampling period: condition factor (F_4, 139 _= 11.619, p < 0.0001), GSI (F_4, 137 _= 19.927, p < 0.0001), HSI (F_4, 139 _= 42.483, p < 0.0001), haemoglobin concentration (F_4, 150 _= 30.747, p < 0.0001), SSI (F_4, 139 _= 8.630, p < 0.0001), leukocyte count (F_4, 150 _= 26.741, p < 0.0001), lymphocyte count (F_4, 149 _= 7.484, p < 0.0001), phagocyte count (F_4, 150 _= 17.871, p < 0.0001), respiratory burst (F_4, 150 _= 14.131, p < 0.0001), IgM concentration (F_4, 149 _= 7.484, p < 0.0001), activity of complement (F_4, 133 _= 29.293, p < 0.0001) and concentration of 11-ketotestosterone measured in males (F_4,84 _= 4.541, p < 0.01).

**Figure 2:**
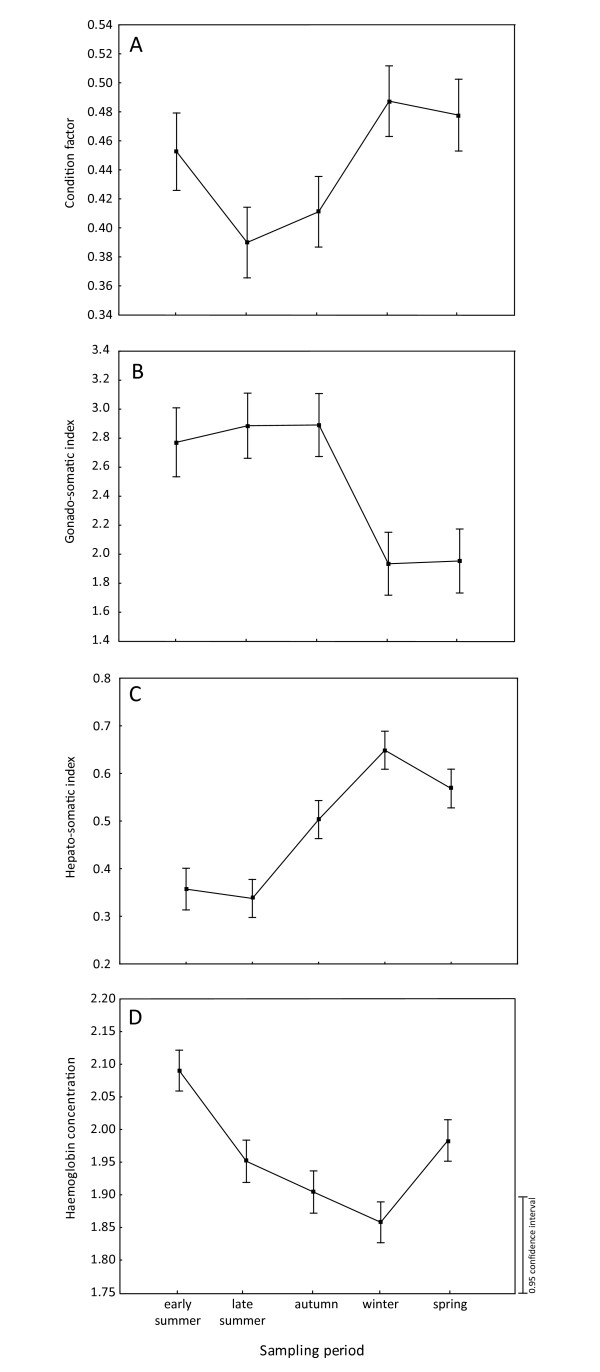
**The changes in physiological variables**. Detailed legend: The changes in the following physiological variables (A) condition factor, (B) gonado-somatic index, (C) hepato-somatic index and (D) haemoglobin concentration in relation to sampling period. Log transformation for condition factor, hepato-somatic index and haemoglobin concentration; and hyperbolic arcsine square root transformation for gonado-somatic index were applied.

**Figure 3:**
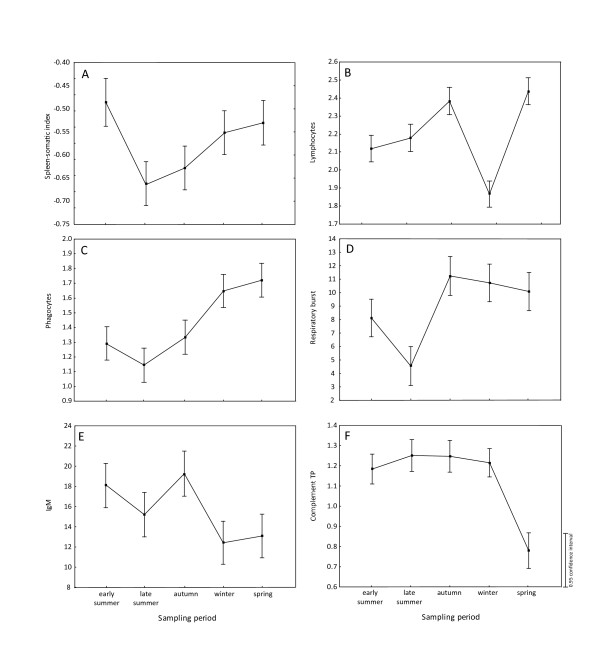
**The changes in immune variables**. Detailed legend: The changes in the following immune variables (A) spleen-somatic index, (B) lymphocyte count, (C) phagocyte count, (D) respiratory burst activity, (E) IgM level and (F) complement activity in relation to sampling period. Log transformation for spleen-somatic index; hyperbolic arcsine square root transformation for phagocyte count, lymphocyte count and complement activity; and square root transformation for respiratory burst were applied.

The highest values of condition factor and HSI were detected in winter and spring (Figure 2A, C), whereas GSI reached the highest values in summer and autumn (Figure 2B). The highest values of haemoglobin concentration (Figure 2D) and SSI (Figure 3A) were found in early summer. Lymphocyte counts (Figure 3B) and total leukocyte counts (not shown) showed similar seasonal variations with high values in autumn and spring and low values in winter. On the other hand, the phagocyte count increased from summer to winter and reached maximum values in spring (Figure 3C). Values of respiratory burst were low in late summer and significantly increased in autumn and the following periods (Figure 3D). The highest IgM concentration level was recorded in early summer and autumn (Figure 3E). The concentration level of 11-ketotestosterone in males increased in spring (not shown), whereas complement activity (considered for both males and females) achieved its lowest values in spring (Figure 3F).

The effect of sex on each immune and physiological variable was tested using ANOVA. Significant differences were revealed only for IgM concentration (F_1,152 _= 34.265, p < 0.0001) with significant higher values in females, and for haemoglobin concentration (F_1, 153 _= 24.588, p < 0.0001) with significant higher values in males.

### Parasitism versus immunity and physiology: the effect of sampling period

**Figure 4:**
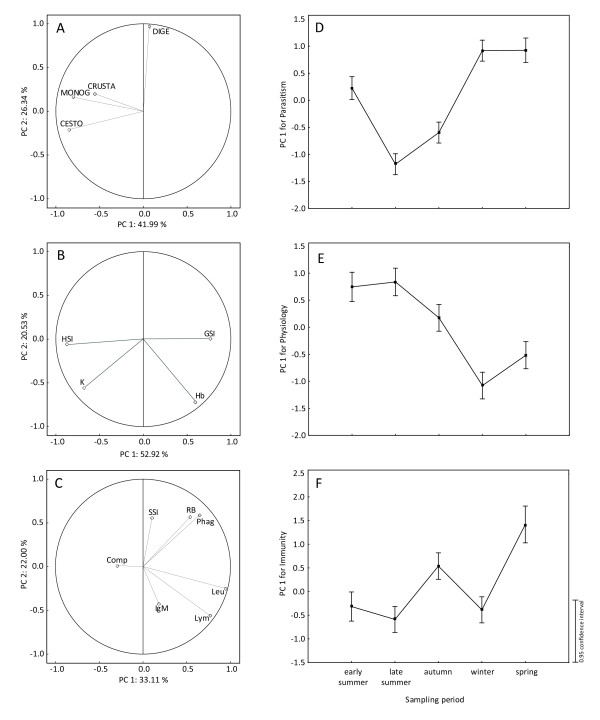
**PCA on parasitism, physiology and immunity in common carp**. Detailed legend: Principal component analyses on (A) parasitism including abundance of Monogenea (MONOG), Crustacea (CRUSTA), Cestoda (CESTO), and Digenea (DIGE), (B) physiological variables including condition factor (K), gonado-somatic index (GSI), hepato-somatic index (HSI), haemoglobin concentration (Hb), and (C) immune variables including spleen-somatic index (SSI), leukocyte (Leu), lymphocyte (Lym) and phagocyte count (Phag), respiratory burst (RB), IgM concentration (IgM) and complement activity (Comp). The changes of (D) index of parasitism (using PC1 of parasitism), (E) index of physiology (using PC1 of physiology) and (F) index of immunity (using PC1 of immunity) in relation to sampling periods are shown.

PCA performed on parasites showed that the first three axes accounted for most of the total variability in the data set. The first axis explained 42.0%, the second explained 26.3% and the third explained 21.1% of the total variability in the parasitological data set (see Figure 4A for the representation using first and second axes). The values of the first three PCs were extracted as a measure of parasitism (PCs 1, 2 and 3 for parasitism). PC1 for parasitism was negatively related to abundance of Monogenea, Cestoda and Crustacea. PC2 for parasitism was positively related to abundance of Digenea and finally, PC3 for parasitism was positively related to abundance of Crustacea and negatively to abundance of Monogenea (Table 2). The PCA on physiology showed that the first three axes accounted for most of the total variability in the physiological data set. The first axis explained 52.9%, the second explained 20.5% and the third axis explained 17.2% of the variability in the data set (see Figure 4B for the representation using first and second axes). The values of the first three PCs were extracted as a measure of physiology (PCs 1, 2 and 3 for physiology). PC1 for physiology was negatively related most clearly to LSI and condition and positively to GSI and haemoglobin concentration. PC2 for physiology was negatively related to haemoglobin concentration and condition. PC3 for physiology was positively related to GSI (Table 2). Finally, we performed similar analyses for the immune variables. PCA showed that the first three axes accounted for most of the total variability in the data set, with the first axis explaining 33.1%, the second explaining 22.0% and the third explaining 16.4% of the variability in the data set (see Figure 4C for the representation using first and second axes). The values of the first three PCs were extracted as a measure of immunity (PCs 1, 2 and 3 for immunity). PC1 for immunity was most clearly related to leukocyte, lymphocyte and phagocyte counts, and respiratory burst. PC2 for immunity was positively related to phagocyte count, respiratory burst and SSI, but negatively related to lymphocyte count. Finally, PC3 for immunity was indicative of the complement system activity and, IgM level (Table 2).

**Table 2:** Component scores of the three principal components of host physiology, host immunity and parasitism.

Physiology	PC1	PC2	PC3
K	**-0.67**	**-0.55**	0.46
GSI	**0.75**	0.00	**0.60**
LSI	**-0.87**	-0.07	-0.07
Haemoglobin	**0.60**	**-0.72**	-0.34
Cumulative R^2^	*52.92*	*73.45*	*90.62*

Immunity	PC1	PC2	PC3

SSI	0.10	**0.56**	-0.11
Leukocyte count	**0.94**	-0.25	-0.04
Respiratory burst	**0.54**	**0.57**	0.41
Complement	-0.30	0.01	**0.76**
IgM concentration	0.18	-0.43	**0.61**
Lymphocyte count	**0.77**	**-0.56**	-0.10
Phagocyte count	**0.65**	**0.59**	0.03
Cumulative R^2^	*33.11*	*55.11*	*71.52*

Parasitism	PC1	PC2	PC3

Monogenea	**-0.80**	0.16	**-0.40**
Crustacea	**-0.56**	0.20	**0.80**
Cestoda	**-0.85**	-0.21	-0.16
Digenea	0.07	**0.97**	-0.13
Cumulative R^2^	*41.99*	*68.33*	*89.45*

The most important parameters contributing to the principal components are shown in bold.

Sampling period was significantly correlated with PC1 and PC3 for parasitism, PC1 for physiology and PC1 and PC2 for immunity (p < 0.001). The seasonal variation of parasitism, immunity and physiology using PC1 is shown in Figure 4D-F). After correcting for sampling period (Table 3), the significant negative correlation between PC1 for parasitism and two PCs for physiology as well as between PC3 for parasitism and PC2 for physiology were found. Moreover, PC1 for parasitism with PC2 for immunity and PC2 for parasitism with PC1 for immunity were significantly positively correlated. Finally, PC2 for immunity was significantly negatively correlated with PC1 and PC2 for physiology, but PC3 for immunity was significantly positively correlated with PC2 and PC3 for physiology.

**Table 3:** Partial correlations controlling for sampling period.

	PC1 for Parasitism	PC2 for Parasitism	PC3 for Parasitism	PC1 for Physiology	PC2 for Physiology	PC3 for Physiology	PC1 for Immunity	PC2 for Immunity
PC1 for Parasitism	1							
PC2 for Parasitism	0.039	1						
PC3 for Parasitism	**0.221**	-0.070	1					
PC1 for Physiology	**-0.300**	0.058	-0.138	1				
PC2 for Physiology	**-0.464**	0.145	**-0.333**	0.121	1			
PC3 for Physiology	-0.108	-0.043	-0.026	-0.078	-0.011	1		
PC1 for Immunity	-0.045	**0.187**	0.067	0.065	-0.177	0.075	1	
PC2 for Immunity	**0.485**	0.014	0.152	**-0.283**	**-0.194**	-0.101	-0.137	1
PC3 for Immunity	-0.105	0.095	-0.060	-0.099	**0.235**	**0.385**	0.148	0.026

Statistical significant correlations (p < 0.05) are shown in bold.

### The different parasite life strategies: a link with host physiology and immunology

GLM analyses on the abundance of different parasite groups (representing parasites with different life strategies) as a function of immune and physiological variables and taking into account sampling periods were performed (Table 4). Except for the case of Digenea, the abundance of all other analyzed parasite groups (i.e. Monogenea, Cestoda and Crustacea) was found to be significantly dependent on sampling period. Moreover, significant relationships between the abundance of Monogenea and two physiological variables, i.e. condition factor and haemoglobin concentration, were found. The abundance of Cestoda was significantly related to phagocyte count and respiratory burst when taking into account the effects of sampling period and sex (both effects were included in GLM following the results of one-way ANOVA). GLM analyses showed a significant partial relationship between the abundance of Digenea and SSI, although the model was not significant (see Table 4).

**Table 4:** GLM analyses on the relationship between parasite abundance, immunity and physiology

Dependent variable	Independent variables	SS	Df	F	p	Total F (p)
Monogenea	Condition factor	1.653	1	11.144	0.001	
	Haemoglobin	0.588	1	3.966	0.049	
	Sampling	13.619	4	22.958	0.000	22.209 (< 0.0001)
Crustacea	Sampling	1248.215	4	7.114	0.000	5.826 (< 0.0001)
Cestoda	Respiratory burst	5.357	1	5.639	0.019	
	Phagocytes	7.865	1	8.278	0.005	
	Sampling	56.846	4	14.958	0.000	8.841 (< 0.0001)
Digenea	SSI	174.815	1	5.648	0.019	1.683 (0.074)

GLM analyses on the relationship between parasite abundance and immune or physiological variables, taking into account sampling period.

### Associations between host immunity and physiology

Using GLM analyses, associations between host immunity (i.e. SSI, phagocyte count, respiratory burst, complement activity and IgM concentration) and physiology (condition factor, GSI, HSI and haemoglobin) taking into account the effect of sampling period or, alternatively, the effects of both sampling period and sex, were analyzed. All variables of immunity and physiology, except 11-ketotestosterone concentration, appeared statistically dependent on sampling period (Table 5). Significant relationships between condition factor, GSI and HSI were found when taking into account the sampling period. Haemoglobin concentration was related to GSI and affected by both sampling period and sex. In addition, 11-ketotestosterone concentration measured in males was significantly related to gonad weight (measured by GSI) and linked to host immunity measured by respiratory burst and complement activity, which suggests a potential trade-off between immunity and reproduction. The reversed patterns of seasonal changes for 11-KT (not shown) and complement activity (Figure 3F) levels were revealed. Phagocyte counts were related only to respiratory burst (a measure of phagocyte activity). However, no relationships among the different measures of immunity or between immunity and physiology were found when taking into account the sampling period (p > 0.05).

**Table 5:** GLM analyses on the relationship between host immunity and physiology

Dependent variable	Independent variables	SS	Df	F	p	Total F (p)
Condition factor	GSI	0.018	1	4.628	0.034	
	HSI	0.059	1	15.24	0.000	
	Haemoglobin	0.019	1	5.039	0.027	
	Sampling	0.131	4	8.505	0.000	6.629 (< 0.0001)
GSI	Condition factor	1.275	1	4.628	0.034	
	HSI	3.948	1	14.329	0.000	
	Haemoglobin	2.321	1	8.423	0.004	
	IgM	4.237	1	15.378	0.000	
	Sampling	5.853	4	5.311	0.001	11.34 (< 0.0001)
HSI	Condition factor	0.152	1	15.24	0.000	
	GSI	0.143	1	14.329	0.000	
	Sampling	0.514	4	12.916	0.000	20.054 (< 0.001)
Haemoglobin	GSI	0.043	1	7.681	0.007	
	Sampling	0.351	4	15.763	0.000	
	Sex	0.129	1	23.191	0.000	11.384 (< 0.001)
SSI	Sampling	0.44	4	6.446	0.000	3.691 (0.0001)
Phagocytes	Respiratory burst	2.924	1	35.009	0.000	
	Sampling	1.871	4	5.602	0.000	10.771 (< 0.0001)
Respiratory burst	Phagocytes	402.309	1	35.009	0.000	
	Sampling	242.144	4	5.268	0.001	9.922 (< 0.0001)
IgM concentration	Sampling	378.458	4	4.372	0.003	
	Sex	504.633	1	23.316	0.000	
	Sampling_Sex	597.857	4	6.906	0.000	7.773 (< 0.0001)
Complement	Sampling	1.972	4	19.414	0.000	9.189 (< 0.0001)
11-ketotestosterone	GSI	6586.91	1	25.556	0.000	
	Respiratory burst	1791.337	1	6.95	0.011	
	Complement	3611.305	1	14.011	0.000	6.524 (< 0.0001)

GLM analyses on the relationship between immune and physiological parameters, taking into account sampling period and sex effect.

**Figure 5:**
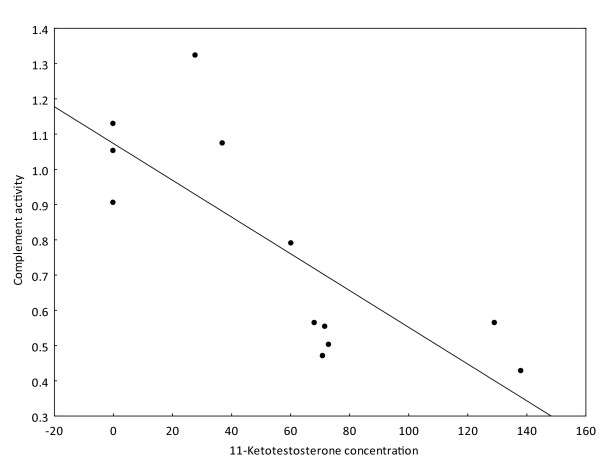
**The negative relationship between the level of 11- ketotestosterone and the activity of complement in spring**.

The last analysis was restricted to the spring season and to males as the level of 11-ketotestosterone was only significantly higher in this period (following the results of ANOVA). A significant negative relationship between 11-ketotestosterone and the level of total complement pathway was found (N = 12, b = -0.78, p > 0.01) suggesting the potential immunosuppression by steroid hormones (Figure 5). There were no significant relationships for any other immune variables in the spring period (p > 0.05).

## Discussion

### The relationship between abiotic environment and parasite infection

Changes in parasite abundance in relation to their life-cycle have been generally considered to be influenced by both host environment and host physiology [2,73]. Differences in the seasonal dynamic of abundance changes among different parasite groups are then predetermined by parasite life-strategies. Moreover, both the presence and efficiency of intermediate hosts play an important role in the transmission of endoparasites (e.g. [74]). In our study, we confirmed that seasonality influence the abundance of Monogenea, Crustacea and Cestoda. Change in water temperature (one of the principal cues of seasonality) is commonly regarded as one of the most important factors determining the presence and abundance of Monogenea [1]. We observed a different seasonal pattern of the abundance changes in oviparous gill parasites of *Dactylogyrus *and *Eudiplozoon *(with maximum abundance observed in summer) compared to viviparous *Gyrodactylus *species (with maximum abundance in winter). *Dactylogyrus *species were the most abundant parasites. The general trend associated with their life-cycle (direct-transmitted ectoparasites) is that any increase in temperature leads to an increase of their population densities [75]. Our results demonstrating the high abundance of all *Dactylogyrus *species of common carp in summer confirmed that water temperature is the main factor determining the high abundance of all *Dactylogyrus *species of common carp.

Adult stages of *Atractolytocestus huronensis *represented the dominant cestode species of common carp in this study. The abundance of cestode infection can be connected with the temporal presence of intermediate hosts. The highest cestode abundance was recorded in late summer. Similar seasonal changes in the abundance of *A. huronensis *were previously reported for German pond-farmed carp [76]. Moreover, this cestode species was the only parasite affected weakly by host sex. Reimchen and Nosil [77], who monitored the level of parasitism in a population of threespined stickleback (*Gasterosteus aculeatus*), showed that females were more likely to be parasitized by the cestode *Schistocephalus solidus *(Cestoda) and suggested that this sex-biased infection could be connected with a dietary niche variation, which may result in differential exposure to infected intermediate hosts.

Digeneans parasitizing common carp were represented mainly by the larval stage (metacercaria) of *Diplostomum *species, which live in the eyes of this second intermediate fish host. This ubiquitous parasite causes cataracts, reduces fish vision, and may even induce total blindness [78]. A marginal effect of seasonality on the abundance of this species was observed. The highest abundance values were found in early summer and autumn. Similar findings have been documented in several studies (e.g. [79,80]), and Burrough [81] suggested that the first peak of infection (early summer) may probably come from the snails that survived through the winter. A second peak of infection may occur in autumn from snails that hatched during the spring period.

Crustacea were represented by an abundant species, *Argulus foliaceus*, which is considered to be an obligate branchiuran ectoparasite infecting many freshwater fish species. Some *Argulus *species are able to tolerate a wide range of water temperatures (e.g. [82]). *Argulus foliaceus*, a common species in Europe, is known to reach high abundance on their hosts during late summer and early autumn (e.g. [83,84]), which was also found here. However, no infection was observed in winter. Hakalahti and Valtonen [85] showed a low abundance of *Argulus coregoni *in fish during winter suggesting that this species can survive winter due to overwintering egg stages laid in autumn.

### The link between host immunity and physiology

We hypothesized that fish investing more in immunity should show less investment in other physiological functions. Moreover, we also hypothesized that seasonality acts as an important factor determining the levels of fish physiology and immunological activity. Using PCA, we revealed that one PC for physiology and two PCs for immunity (from three PCs analyzed) were correlated with seasonality suggesting that the majority of measured variables are under seasonal changes. The different PCs for immunity and physiology were correlated with different variables (mainly when comparing PC1 and 2 to PC3). This fact together with the significant correlations between PCs of immunity and physiology may suggest the potential relationships between different immunity and physiology variables.

We conducted analyses among selected measures of host status related to condition and reproduction and of immunity taking into account the sampling period. Fish store energy in muscle tissues or in the liver (glycogen) during periods of high food and energy intake [86]. Therefore, both condition factor and the relative size of liver (HSI) are recommended as an indirect indicator of energy status [86]. The gonado-somatic index (GSI) represents an accurate assessment of reproductive maturity. Negative relationships were found between fish energy status (measured either by the condition factor or HSI) and GSI, suggesting the existence of a trade-off, with a decreasing fish condition in the period of gonad formation and reproduction. A significant relationship was also observed between haemoglobin concentration and GSI. The seasonal variation in haemoglobin concentration is potentially related to variation in water temperature and variation in water oxygen concentration. Fish adapt via increases in total haemoglobin concentration or by other mechanisms such as changes in red cell nucleoside triphosphate concentration [87,88]. The spawning process may affect haematological parameters [89], which may explain the observed relationship between haemoglobin concentration and GSI. Moreover, the concentration of haemoglobin was the single physiological parameter that differs between females and males, which has already been observed in other fish species [51].

Although significant relationships were found between PCs of physiology and immunity suggesting the potential trade-off associations between physiology and phagocyte activity or SSI (using PC2 for immunity and PCs 1 and 2 for physiology) and between physiology and complement activity or IgM (using PC3 for immunity and PCs 2 and 3 for physiology), no associations between individual immune variables and physiological variables were found using GLM. However, host immunity was strongly dependent on the sampling period, which confirms that seasonality is the driving force of immune variation in fish.

We hypothesized that higher investments in somatic condition could be associated with lower investments in immune defence. Although, our analyses revealed no significant relationship between condition factor and spleen size, both these variables showed similar seasonal dynamics. The highest values of SSI were found in early summer followed by decreasing values in late summer. Our findings are not in agreement with previous studies in roach *Rutilus rutilus *[90] or in Arctic charr, *Salvelinus alpinus *[91], where spleen size was shown to decrease in the breeding season.

Concentration levels of IgM were also dependent on the sampling period with low IgM values recorded in winter and spring. Previous studies on IgM concentration showed a decrease in IgM level during the winter period, probably related to water temperature, in rainbow trout *Oncorhynchus mykiss *[46] and goldfish *Carassius auratus *[92]. According to Avtalion [93] and Stolen et al. [94], low water temperature causes a selective suppression of in vitro T cell responses and antibody synthesis. Suzuki et al. [95] followed the annual changes of IgM in three strains of rainbow trout under constant water temperature and natural day length, and showed that the IgM concentration level varied due to the immunosuppression effect induced by sex hormones. Here, we did not find any clear association between seasonal changes (potentially related to water temperature) and IgM concentration level. The lowest water temperature was recorded in autumn and winter whilst the lowest concentration in IgM level was observed in winter and spring. Moreover, IgM concentration was the only immune variable affected by host sex, with a significantly lower IgM concentration in males. Although, the immunosuppressive effect of testosterone and/or gonad maturation on IgM has already been demonstrated in rainbow trout [96], this effect has not been investigated in common carp before our study. Other hormones like cortisol may also reduce the IgM secretion, as shown in salmonid fish [97].

The annual fluctuation in complement activity was investigated in gilthead sea bream, *Sparus aurata *by Hernández et al. [7]. These authors showed increasing complement activity in warm months probably in relation to higher metabolic activity at higher temperatures in poikilothermic organisms. In this study, the total complement activity decreased in spring but not in winter at the coldest temperature. Seasonal changes (potentially related to water temperature) does not seem to affect the total complement activity and its decrease in spring could be related to reproduction (see below its relationship with 11-ketotestosterone).

Phagocyte counts and phagocyte activity (measured by the respiratory burst) were both affected by seasonality, although these variables were highly correlated. Seasonal variability in phagocyte activity has been investigated in several fish species with inconsistent conclusions. An immunosuppressive effect of water temperature on the innate immune response in catfish *Ictalurus punctatus *[11] and in tench, *Tinca tinca *[98,99] has been observed. In accordance with these studies, we recorded low values of respiratory burst in late summer, where water temperature is higher comparing to other period investigated. High values of respiratory burst were recorded from autumn to spring. Phagocyte activity seems not to be suppressed by water temperature in several fish species e.g. rainbow trout under laboratory conditions [35].

11-ketotestosterone is considered to be a major androgen hormone in teleost fish (see review by Borg [50]). This hormone influences spermatogenesis and effects the expression of secondary sexual characters and reproductive behavior. It also suppresses several immune functions. Steroid hormones have dualistic functions: they increase the expression of elaborated sexual ornamentation but decrease the immunocompetence of an individual. This observed mechanism is at the basis of the immune-handicap hypothesis [28]. We found a positive association between gonad size (measured by GSI) and 11-ketotestosterone concentration. Moreover, we found support for the immunosuppressive role of 11-ketotestosterone, with negative relationships observed between the concentration of 11-ketotestosterone and two immune parameters - total complement activity and respiratory burst (taking into account the sampling period). Using linear regression (not included in the results), only complement activity was negatively correlated with concentration of 11-ketotestosterone. A significant immunosuppressive effect of 11-ketotestosterone on complement activity was also found when using only spring data, i.e. where concentrations of 11-ketotestosterone were the highest and correlated with gonad development.

### The role of the season: host immunity and physiology versus total parasite load

One aim of our study was to investigate the potential link between parasitism and host immunity or physiology on two different scales. First, using the computed PCs we showed the significant role of seasonality on each of parasitism, immunity and physiology.

After correcting for sampling period, the negative relationships between parasitism and physiology were found, which suggests that a "good" physiological status reflects a host's ability to escape from parasitism (especially concerning Monogenea, Crustacea and Cestoda). Moreover, parasitism was positively related to immunity, which could indicate that despite of strong effect of seasonality on fish immunity, this system (or at least some immune pathways) is activated by increasing level of parasite infection.

### Parasitism versus host immunity and physiology: effect of season or a real association?

Effects of seasonal variability on the parasite abundance of several parasite groups that differ according to life strategy were observed. A significant relationship between monogeneans, the most abundant ectoparasite group, and the condition of fish was found, taking into account the sampling period. The opposite seasonal patterns in fish condition and in the abundance of monogeneans (Figures 1A and 2A) suggest that high infection by these parasites should be detrimental to fish.

A negative relationship between haemoglobin concentration and the same parasites was also observed. The presence of *Eudiplozoon nipponicum*, a haematophagous monogenean species with intracellular blood digestion [100] and a body size 25-60 times higher than the average body size of *Dactylogyrus *and *Gyrodactylus *species, may contribute to the decrease in haemoglobin concentration. Sitja-Bobadilla and Alvarez-Pellitero [101] suggested that even a low intensity of infection by a monogenean species (*Sparicotyle chrysophrii*) may induce fish anaemia, and activation of fish haematopoesis leading to an increase in immature erythrocytes with lower amounts of haemoglobin.

We found a significant relationship between the abundance of Cestoda and counts of peripheral blood phagocytes. Moreover, the immunological activity of phagocytes measured by the respiratory burst was also found to be associated with cestode infection (taking into account the sampling period). The abundance of cestodes (*A. huronensis *was the dominant species) was negatively related to both these measures of immunity using linear regression (not shown in the results section). We could hypothesize that, during a period of low parasite infection, phagocytes are present in peripheral blood ready to react quickly to an entering antigen derived from parasites. However, during high parasite infection, the phagocytes and other blood cells colonize the tissues surrounding the attachment organs of *A. huronensis *[102]. A low activity of phagocytes, measured by respiratory burst, was also demonstrated in fish experimentally infected by the cestode *Schistocephalus solidus *[103] or in gynogenetic form of triploid *Carassius auratus *infected by metacercaria of *Metagonnimus *sp. [104]. Other examples demonstrating the depression of oxidative burst and/or the impairment of phagocyte activity induced by parasites are given by Alvarez-Pellitero [19]. In addition, some parasites are able to exploit the host immune reaction in order to improve their attachment to the host tissue. Alvarez-Pellitero [19] documented that attachment of the cestode *Cyathocephalus truncatus *in the fish pyloric caeca was facilitated by an inflammatory reaction. However, Vainikka et al. [72] observed the positive correlation between parasite loads and the relative proportion of phagocytes in blood of roach (*Rutilus rutilus*). They also showed that functional characteristics of these cells were positively related to the proportion of dead *Rhipidocotyle campanula *(Digenea) which may indicate that the chemiluminescence method is a suitable measure to estimate functional immunocompetence in fish.

A significant relationship was observed between the abundance of digeneans and SSI. However, using simple linear regression (not shown in the results section), a positive relationship was found between larval digeneans of *Diplostomum *species and relative spleen size. Skarstein et al. [90] suggested that large spleen in fish may reflect the ability to respond to parasite infection or may indicate high immunological activity against already established infection. The associations between spleen size and parasitism by metazoan parasites have been tested in many intraspecific studies (e.g. [29,59]), but a significant relationship is rarely reported [e.g. [30,61]]. Vainikka et al. [72] did not find any associations between spleen size and parasite counts in roach suggesting that spleen size might not represent the measure of immunocompetence in roach and thus this variable should be interpret with caution in immunoecological studies. Moreover, they suggest that it is difficult to interpret causal relationships when using only correlation study to analyze the associations between immune variables and parasite load and therefore, they propose that the experimental studies are needed.

## Conclusions

Our study showed that host immunity and physiology, as well as parasite infection, are highly dependent on seasonal variability (i.e. temporal variation) potentially related to the changes of water temperature (one of the principal cues of seasonality), although several other abiotic characteristics of the water environment may play a part. Nevertheless, we confirmed the associations between parasitism and both host physiology and immunity after correction for temporal variability. When considering parasites with different life strategies, and taking into account the effects of seasonality, fish in a worse physiological condition suffer from a higher level of infection by the abundant ectoparasitic monogeneans. The infection by cestodes seems to activate several mechanisms of the immune system and particularly phagocyte activity. Seasonal variability affects host immunity and physiology through energy allocation in a trade-off between important functions, i.e. reproduction and fish condition. However, the measures of host immunity were not found to be in a direct trade-off with the investigated physiological traits or functions, but the immunosuppressive role of 11-ketotestosterone was observed.

## Competing interests

The authors declare that they have no competing interests.

## Authors' contributions

AŠ designed this study. KR and AŠ drafted the manuscript. SM significantly contributed in drafting the statistical part of manuscript. SM, MF and PH involved in revising for important content and discussing the results. All authors contributed to acquisition of data, data analysis or data interpretation. All authors read and approved the final version of the manuscript.

## Acknowledgements and Funding

This study was funded by the Grant Agency of the Czech Republic, project No. 524/07/0188. MF was also supported by the Ministry of Education project MSM6007665809. KR was funded by the Ichthyoparasitology Research Centre of the Ministry of Education, Youth and Sports of the Czech Republic LC 522 and partially by the Rector's Programme in Support of MU Students' Creative Activities. AŠ was supported by the Research Project of Masaryk University (No. MSM0021622416)
